# Preoperative Treatment Expectations and Their Association with Postoperative Quality of Life and Patient Satisfaction in Non-Orthopedic Surgery: A Systematic Review

**DOI:** 10.3390/ijerph23060804

**Published:** 2026-06-16

**Authors:** Nanna W. Christiansen, Thora G. Thomsen, Elizabeth E. Rosted, Marianne Krogsgaard, Marian C. Petersen, Anja Geisler

**Affiliations:** 1 Department of Anesthesiology, Zealand University Hospital, 4600 Koege, Denmark; nanchr@regionsjaelland.dk; 2 Department of Ophthalmology, Zealand University Hospital, 4000 Roskilde, Denmark; thst@regionsjaelland.dk; 3 Department of Clinical Oncology and Palliative Care, Zealand University Hospital, 4000 Roskilde, Denmark; eros@regionsjaelland.dk; 4 Department of Regional Health Research, University of Southern Denmark, 5230 Odense, Denmark; 5 Center for Surgical Science, Department of Surgery, Zealand University Hospital, 4600 Koege, Denmark; markro@regionsjaelland.dk (M.K.); mapete@regionsjaelland.dk (M.C.P.); 6 Department of People and Technology, Roskilde University, 4000 Roskilde, Denmark; 7 Department of Transplantation and Organ Surgery, Copenhagen University Hospital, Rigshospitalet, 2100 Copenhagen, Denmark; 8 Department of Clinical Medicine, Copenhagen University, 2100 Copenhagen, Denmark

**Keywords:** patient expectations, satisfaction, quality of life, preoperative expectations, surgery, patient-reported outcomes, person-centred care

## Abstract

**Highlights:**

**Public health relevance—How does this work relate to a public health issue?**

- Preoperative expectations shape patients’ physiological responses to surgery, influencing pain perception, anxiety, coping, and overall recovery. Because these responses affect postoperative outcomes at the population level, understanding expectations is directly relevant to improving the quality of surgical care and reducing avoidable suffering.
- Misaligned or unmet expectations can undermine patient well-being, satisfaction, and sense of control, particularly when postoperative realities differ from what patients anticipated. This creates a public health concern, as inadequate communication and expectation management can contribute to poorer recovery trajectories, increased healthcare utilization, and disparities in patient experience.

**Public health significance—Why is this work of significance to public health?**

- Identifying how preoperative expectations affect quality of life and satisfaction in non-orthopedic surgical patients fills a critical evidence gap, enabling health systems to design more effective, patient-centered communication strategies that support psychological readiness and reduce postoperative distress.
- Improving expectation alignment through tailored, empathetic communication has the potential to enhance recovery, reduce anxiety, and strengthen trust in healthcare, ultimately contributing to better population-level surgical outcomes and more equitable patient experiences across diverse surgical pathways.

**Public health implications—What are the key implications or messages for practitioners, policy makers and/or researchers in public health?**

- When preoperative expectations are addressed in a patient-centered manner, clinicians may help align patients’ anticipated outcomes with what is realistically achievable.
- This process can strengthen patient empowerment and potentially prevent possible nocebo effects.

**Abstract:**

Background: Patients’ preoperative expectations may influence postoperative quality of life and satisfaction. Aim: To provide an overview of the evidence regarding the association between patients’ preoperative expectations and postoperative quality of life as the primary outcome, with postoperative satisfaction included as a secondary outcome. Methods: A systematic review was conducted in accordance with the PRISMA guidelines and Cochrane Handbook of Systematic Reviews guidelines. A comprehensive search was conducted across five databases for all study types. Results: Ten cohort studies met the inclusion criteria, encompassing 1013 patients undergoing various surgical procedures. Most studies exhibited a high risk of bias, and several employed unvalidated instruments to assess preoperative expectations. Only one study found a significant association between preoperative expectations and quality of life. One study reported significantly better perceived health among patients with high preoperative expectations, and four studies identified a significant relationship between preoperative expectations and post-operative satisfaction. Overall, the findings were inconsistent, and the included studies demonstrated substantial methodological heterogeneity. Conclusions: Only individual studies identified significant associations with quality of life, perceived health, or postoperative satisfaction; overall, the findings were inconsistent. The evidence is limited by high risk of bias, heterogeneous methodologies, and use of non-validated measurement tools. Further research is needed to clarify the role preoperative expectations have in postoperative outcomes.

## 1. Introduction

Preoperative expectations (PE) are increasingly recognized as an important factor influencing how patients anticipate and interpret healthcare experiences [1]. Expectations are future-oriented cognitive constructs formed through prior experiences, interactions with healthcare professionals, social context, and broader cultural narratives about illness and recovery [2,3]. As such, expectations operate as cognitive schemas that help patients interpret forthcoming events and manage the inherent uncertainty of surgical treatment. Within psycho-neurobiological research, expectations are understood as a core mechanism underlying placebo and nocebo effects [4,5]. Positive expectations can enhance perceived treatment benefit, reduce, e.g., pain, and improve coping, while negative expectations may exacerbate symptoms, anxiety, and dissatisfaction [4,5]. These findings emphasize that expectations do not merely reflect passive beliefs but actively modulate physiological and psychological responses to surgery. According to Weick [6], sensemaking is triggered when individuals encounter ambiguity or unexpected events, prompting them to reinterpret and reconstruct meaning. In the surgical context, when postoperative realities diverge from preoperative expectations—such as greater pain, slower recovery, or unanticipated complications—patients engage in sensemaking to reconcile these discrepancies. Insufficient or inconsistent information from clinicians can hinder this process, contributing to heightened uncertainty, anxiety, or perceived loss of control [6,7]. Person-centered communication plays a crucial role in shaping expectations and supporting patients’ sensemaking processes. Clear, empathetic, and individualized communication that explores patient concerns, clarifies uncertainties, and provides tailored information has been shown to foster trust, improve psychological readiness for surgery, and contribute to more positive postoperative evaluations [8,9,10], but also helps align expectations with realistic surgical outcomes, reducing the risk of disappointment, enhancing satisfaction [11,12] and quality of life (QoL) [1,13]. QoL is a multidimensional and inherently subjective construct encompassing physical, psychological, emotional, and social dimensions of well-being [14]. In surgical research, QoL is frequently used as a patient-reported outcome reflecting the broader impact of treatment, physical function, pain, and satisfaction with life itself [15]. However, the issue remains complex and somewhat ambiguous, as perceptions of QoL may vary over time and depending on personal values, expectations, coping resources, and sociocultural context [16,17]. While PE has been thoroughly investigated and also associated with postoperative clinical outcomes in orthopedic surgery [18,19,20], evidence regarding other surgical procedures remains limited. Therefore, the aim of this systematic review is to examine the literature according to the association between patients’ PE and postoperative quality of life as the primary outcome and satisfaction as a secondary outcome in non-orthopedic surgical patients.

## 2. Methods

This systematic review followed the Preferred Reporting Items for Systematic Reviews and Meta-Analyses [21] (PRISMA) guidelines and Cochrane as framework [22]. Before initiating the review process, a protocol with registration number CRD42024541544 was registered in the Prospective Register of Systematic Reviews (PROSPERO). The review question guiding this systematic review was: what evidence exists regarding the influence of preoperative expectations on postoperative quality of life and patient satisfaction in non-orthopedic surgical patients?

### 2.1. Data Sources and Search Strategy

A search string was executed in Embase, CENTRAL, CINAHL, PsycINFO, and MEDLINE. Furthermore, the first 500 hits on Google Scholar were included in the screening process to identify potentially relevant studies not indexed in the selected databases and to increase search sensitivity. Google Scholar results were sorted according to relevance, as determined by the platform’s default algorithm, and titles and abstracts were screened consecutively in the order presented. In addition, the reference lists of included studies and relevant reviews were screened manually to identify any further eligible studies. The included studies were categorized according to study design, sample size, type of surgery, follow-up period (3 or 6 months), preoperative expectations, quality of life, and patient satisfaction.

The first author developed and performed the literature search and search string in cooperation with a professional search specialist (librarian) at Zealand University Hospital in Denmark. For an overview of the search string, please see Supplementary Materials S1. The last search was conducted on 5 January 2026. All studies published in English or Nordic languages that investigated patients’ experiences (aged > 18 years) were included. Eligible studies were those that assessed patients’ preoperative expectations using a patient-reported outcome measurement tool or patient interviews conducted within 30 days prior to surgery, and measured QoL or satisfaction with the treatment postoperatively. The exclusion criteria included conference abstracts, correspondence, comments, case studies, protocols, reviews, unpublished observations, and studies conducted in non-hospital settings. Furthermore, studies assessing preoperative expectations (PE) postoperatively were excluded. Notably, orthopedic surgical patients were excluded from this review due to the existence of multiple systematic reviews exploring the relationship between PE and postoperative satisfaction or QoL [13,18,19,20,23] comprising a total of 131 included studies. Furthermore, differences in postoperative risks, recovery patterns, pain management protocols, infection profiles, and rehabilitation pathways would likely require a separate synthesis. If orthopedic patients had been included, subgroup analyses or separate syntheses would therefore have been considered appropriate.

### 2.2. Outcomes

The primary outcome was to assess an association between patients’ preoperative expectations and QoL at three or six months postoperatively. The secondary outcomes were differences in preoperative QoL and QoL measured at three or six months postoperatively, whether preoperative patient expectations affect patient satisfaction at three or six months postoperatively, and patient levels of satisfaction at three or six months postoperatively.

### 2.3. Data Extraction and Quality Assessment

The team of reviewers consisted of six researchers (AG, E.E.R, MK, M.C.P, N.W.C, TT). Title and abstract were independently screened by two authors; the primary investigator (N.W.C) screened all studies and (AG, E.E.R, MK and T.G.T) assisted as second reviewer and extracted an equal part of the studies. Studies deemed eligible by the predefined inclusion and exclusion criteria underwent full-text review, independently by two authors. Any conflicts were solved by the senior author (AG). Covidence systematic review software was used to conduct title, abstract, and full-text screening and to ensure that duplicates were removed (Veritas Health Innovation, Melbourne, Victoria, Australia). Available at www.covidence.org © 2024. Covidence.org is free of charge for those working at Zealand University Hospital, Denmark. The final data extraction was performed in Excel independently by two authors (the primary investigator and one co-author) and compared for discrepancies. The extracted data included author, country, year of publication, study design, study characteristics (surgical procedure, number of included participants, follow-up period), and demographic characteristics.

### 2.4. Study Risk of Bias Assessment

Since only cohort studies were included, the ROBINS-I tool Version 1 was used for bias assessment [24]. The tool comprises the following seven domains: bias due to confounding, selection of participants, deviations from intended interventions, missing data, bias in intervention classification, measurement of outcomes, and selection of the reported result. N.W.C and AG independently assessed the risk of bias, then compared and discussed any discrepancies.

## 3. Results

### 3.1. Study Selection and Characteristics

**Figure 1:**
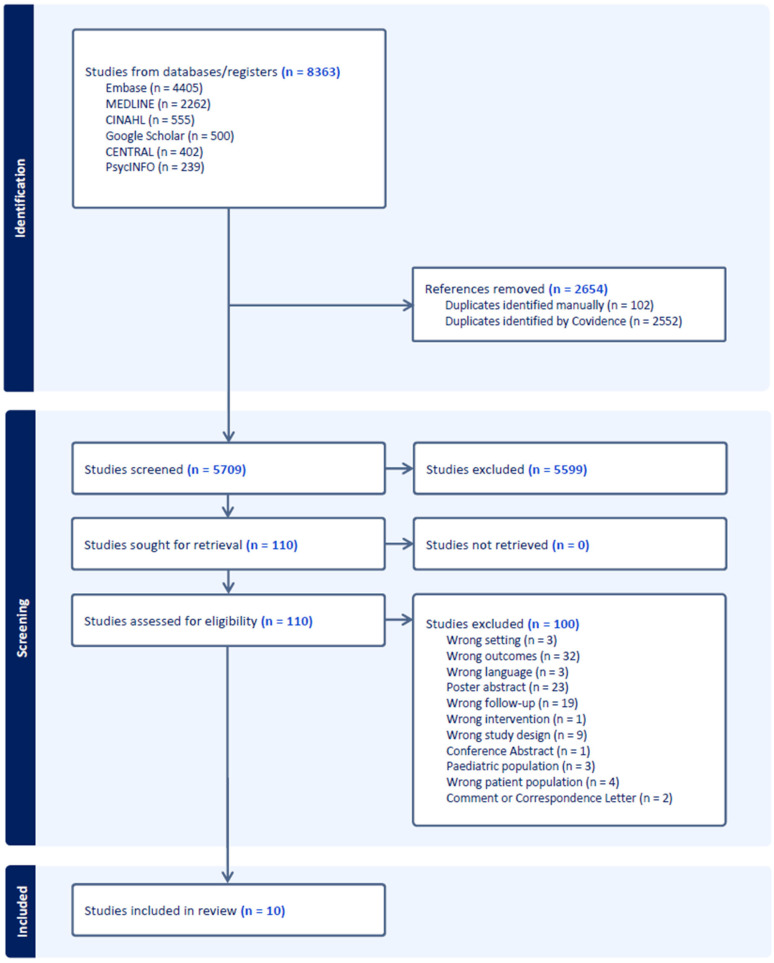
Flowchart of included studies.

Initially, 8363 studies were identified and screened, and 2654 were removed due to duplication. All included studies were published in English. No eligible studies were identified during the screening process in Nordic languages (Swedish, Norwegian, or Danish). After title and abstract screening, 103 studies were retained for full-text review. Of these, ten studies [25,26,27,28,29,30,31,32,33,34] were ultimately included for data extraction (Figure 1). The ten included studies [25,26,27,28,29,30,31,32,33,34] were published between 1995 and 2025, included 1013 patients, comprising 520 females and 493 males. Follow-up periods ranged from two weeks to 24 months. Five studies assessed QoL outcomes [25,26,27,29,34], and five included patient satisfaction [28,30,31,32,33]. For the characteristics of the included studies, see Table 1. The key findings of each included study are shown in Table 2.

### 3.2. Type of Surgery

The studies included the following surgical procedures. Heart transplantation [34], hematopoietic stem cell transplantation [26], kidney transplantation [27,33], cataract surgery [28], sinus surgery [31], bilateral prophylactic mastectomy [25], immediate-loaded zygomatic implant-supported fixed rehabilitation [32], mandibular implant overdentures and conventional complete dentures [30], and bilateral subthalamic deep brain stimulation [29].

### 3.3. Measurements

#### 3.3.1. Preoperative Patient Expectations

Brandberg et al. [25] assessed PE across six life areas, and patients indicated whether they had positive or negative expectations for each area. In the study by Heydecke et al. [30], a questionnaire comprising two self-constructedquestions was used, each rated on a 100 mm Visual Analog Scale (VAS). In the study by Lee et al. [26], pre-transplantation expectations of treatment success were classified a priori based on responses to two questions. Patients rated their agreement using a 5-point Likert scale. In the study by Leedham et al. [34], a positive expectations subscale was created by combining seven items from the self-report QoL Scale. Patients provided ratings on a 7-point Likert scale according to expectations. In the study by Maier et al. [29], semi-structured interviews were used, covering 13 areas, including improvements in motor symptoms, QoL, medication reduction, and walking. The study by Mattos et al. [31] used a self-constructed, unvalidated questionnaire and interviews covering 11 domains, and patients rated their responses using both 5- and 3-point Likert scales. In the study by Mönestam et al. [28], a single-item self-constructedquestionnaire and a 4-point Likert scale were used. The study by Rodrigues et al. [32] used a 100 mm VAS and a self-constructed 5-item questionnaire. In the study by Schoot et al. [33], four items from the 12-item Short Form Survey were used, and the questions were rephrased from an expectancy perspective. The study by Schulz et al. [27] used a questionnaire with three self-constructeditems to assess patients’ expectations of their post-transplant physical, psychological, and social quality of life before transplantation. Three studies [26,29,32] categorized patients into groups based on their level of optimism, classifying them as negative/less optimistic, neutral, medium, or positive/optimistic (Table 3). All tools used to assess PE in the included studies were non-validated.

#### 3.3.2. Quality of Life

This outcome was reported in five studies [25,26,27,29,34]. Brandberg et al. [25] assessed QoL preoperatively and at 6- and 12-month follow-up using the validated Short Form 36 (SF-36). No difference in QoL between the three assessment points was found. Lee et al. [26] used the validated SF-36 and the validated Spitzer Quality of Life Index, preoperatively and six months postoperatively. None of the measures found a significant difference between the two assessment points. Leedham et al. [34] used the non-validated Quality of Life Scale preoperatively, three and six months postoperatively. They found patients’ self-reported QoL to be moderately poor before surgery and increased significantly over time (*p* < 0.001). Maier et al. [29] used the validated Parkinson’s Disease Questionnaire-39 preoperatively and at three month follow-up. They did not find a significant difference between the two assessment points. Schulz et al. [27] rated the physical, psychological, and social dimensions of QoL separately on a 10-point visual analog scale (VAS), anchored at 1 (worst imaginable QoL) and 10 (best imaginable QoL) preoperatively, three, six, and 12 months postoperatively (Table 1). The results show that QoL increased in all dimensions, and improvements were maintained across all assessments except for psychological QoL at the 12-month assessment point.

#### 3.3.3. Postoperative Satisfaction

This outcome was reported in five studies [28,30,31,32,33]. In the study by Heydecke et al. [30], patients’ general satisfaction was measured using a 100 mm Visual Analogue Scale (VAS) at 3 months postoperatively. Mattos et al. [31] measured postoperative patient satisfaction with the surgical outcome using a two-item, self-constructedquestionnaire administered through qualitative interviews at six months post-surgery. In the study by Mönestam et al. [28], satisfaction was assessed five to six months postoperatively using a single-item self-constructedquestionnaire with a 3-point Likert scale. Rodrigues et al. [32] assessed patients’ overall satisfaction regarding aesthetics, speech, and social life at 6 months postoperatively. In the study by Schoot et al. [33] treatment satisfaction was assessed using the Renal Treatment Questionnaire (RTSQ), a 13-item instrument developed for kidney patients to evaluate satisfaction or dissatisfaction with treatment. The total score ranges from 0 to 78, with higher scores indicating greater treatment satisfaction. Decision regret was also assessed using the Decision Regret Scale, a 5-item questionnaire.

#### 3.3.4. Primary Outcome

The primary outcome, the association between patients’ preoperative expectations and their QoL at three or six months postoperatively, was reported in two studies [26,29]. The findings showed contrasting results. In the study by Lee et al. [26], no significant difference was found between the ‘Optimistic’ and ‘Less Optimistic’ groups in QoL at six months postoperatively. However, baseline assessments using the SF-36 revealed that patients with higher expectations had better health perceptions in several QoL domains, including General Health (*p* = 0.003), Social Functioning (*p* = 0.01), Role-Emotional (*p* = 0.02), and Mental Health (*p* < 0.0001) compared with less optimistic patients. The study by Maier et al. [29] divided patients into the following groups: negative, mixed, and positive expectations. When comparing the preoperative scores with the 3-month postoperative scores, QoL improved significantly in the mixed and positive groups. There was a statistically significant difference in QoL between the negative group and the mixed group preoperatively and at 3 months postoperatively, as well as between the negative and positive groups (*p* = 0.001). For an overview of the classification of patients into groups according to expectations please see Table 3.

#### 3.3.5. Secondary Outcomes

##### Differences in Quality of Life Measured Preoperatively and at Three or Six Months Postoperatively

Three studies investigated this outcome [25,27,34]. In the study by Brandberg et al. [25], no significant changes between preoperative QoL and QoL at six months postoperatively were observed. The study did not examine whether there was a correlation between positive or negative PE and postoperative QoL. The study by Leedham et al. [34] reported that patients who rated their preoperative QoL as moderately poor experienced significant improvement over time. QoL increased immediately after surgery and remained elevated at three and six months postoperatively. However, no correlation between PE and QoL was found. In the study by Schulz et al. [27], significant changes in QoL across assessments were reported for all three dimensions: Physical (*p* < 0.001), psychological (*p* < 0.05), and social QoL (*p* < 0.001). Compared to preoperative measurements, QoL increased across all dimensions, with improvements maintained at all assessment points at three, six, and 12 months post-surgery (*p* < 0.05), except for psychological QoL (*p* > 0.05) at 12 months post-surgery. Post-transplant QoL was consistently lower than expected across all dimensions and assessment points. They also found that the interaction between optimistic preoperative expectations and social QoL overestimation at 3 months was significantly associated with distress at 6 months post-surgery. Patients with low optimism reported higher distress after social QoL overestimation (*p* < 0.001).

#### 3.3.6. Preoperative Expectations Related to Postoperative Satisfaction

Five studies [28,30,31,32,33] investigated this outcome. In the study by Heydecke et al. [30], posttreatment satisfaction ratings for two-implant-supported mandibular overdentures (IOD) were numerically higher than pretreatment expectations. However, the differences did not reach statistical significance. A significant association between pretreatment expectations and posttreatment satisfaction was observed only in the Middle-Aged group (35–65 years), whereas no such association was found in the Senior group (65–75 years). Mattos et al. [31] performed a mediation analysis using generalized structural equation modeling (GSEM) with bootstrapping. Their results demonstrated that the fulfillment of preoperative expectations significantly mediated the relationship between clinical outcome and satisfaction (*p* = 0.016), accounting for 34% of the total effect (RIT = 0.34). Notably, the analysis indicated complete mediation, as the direct impact of clinical outcome on satisfaction was no longer statistically significant after controlling for expectation fulfillment.

In the study by Mönestam et al. [28], no correlation was found between high PE and postoperative satisfaction. Patient satisfaction was positively associated with better functional outcomes of the operated eye. Completely satisfied patients exhibited a greater mean improvement in visual acuity (VA) of the operated eye than partially satisfied patients (*p* < 0.05). The improvement index was significantly higher for completely and partially satisfied patients compared to dissatisfied (*p* < 0.001 and *p* < 0.005). Rodrigues et al. [32] reported that a regression model using generalized estimating equations (GEE) based on continuous VAS scores showed that overall satisfaction, as well as satisfaction with aesthetics, speech, and social life, remained stable throughout the entire follow-up period (*p* > 0.05). A statistically significant association was found between preoperative expectations and satisfaction with the rehabilitation’s impact on speech (*p* < 0.001), aesthetics (*p* = 0.007), chewing (*p* < 0.001), and social life (*p* < 0.001). However, regarding changes in satisfaction over time, only chewing showed a statistically significant association at 6 months post-surgery (*p* = 0.025). In the study by Schoot et al. [33], treatment satisfaction was generally high. At 6-month follow-up, data was available for 91% of participants, and the median (IQR) RTSQ score among kidney transplant recipients was 74 (66–78), where 78 represents the highest possible level of satisfaction. No statistical analyses were performed to examine the association between preoperative expectations and postoperative patient satisfaction.

#### 3.3.7. Other Outcomes

Some of the included studies also reported on physical [34] and mental health (apathy, depression, mania, anxiety) [25,26,29]. In the study by Brandberg et al. [25], The Hospital Anxiety and Depression Scale revealed that anxiety decreased over time (*p* = 0.0004) when looking at each assessment point. However, no statistically significant differences were detected for depression. In the study by Lee et al. [26], analyses indicated no significant differences in bothersome symptoms such as anxiety, depression, pain, or difficulty concentrating, nor in the occurrence of depressive syndromes. The Leedham et al. [34] study investigated the relationship between PE and physical health. Six months posttreatment, higher PE scores were associated with better health outcomes, as measured by the Patient Functioning Subscale (r = 0.51, *p* < 0.05). In the Maier et al. [29] study, a one-way ANOVA revealed significant group differences in Apathy Evaluation Scale and Beck Depression Inventory-2 scores at baseline and 3-month follow-up (3 mFU), using a corrected significance level of 0.0125 (0.05 divided by 4 for both time points). Post hoc Bonferroni tests confirmed that apathy scores were significantly higher in the group-neg than the group-mix and group-pos at baseline and 3 mFU. Similarly, depression scores were significantly higher in the group-neg compared with group-mix at baseline and higher compared with both group-mix and group-pos at 3 mFU. State anxiety, as measured by the State-Trait Anxiety Inventory, was higher in the group-neg compared with the group-mix at baseline and higher in the group-neg compared with the group-mix and group-pos at 3 mFU. No significant differences were observed for the Self-Report Manic Inventory at baseline or 3 mFU. Depression scores significantly improved at 3 mFU, but only within the group-pos.

### 3.4. Risk of Bias

**Figure 2:**
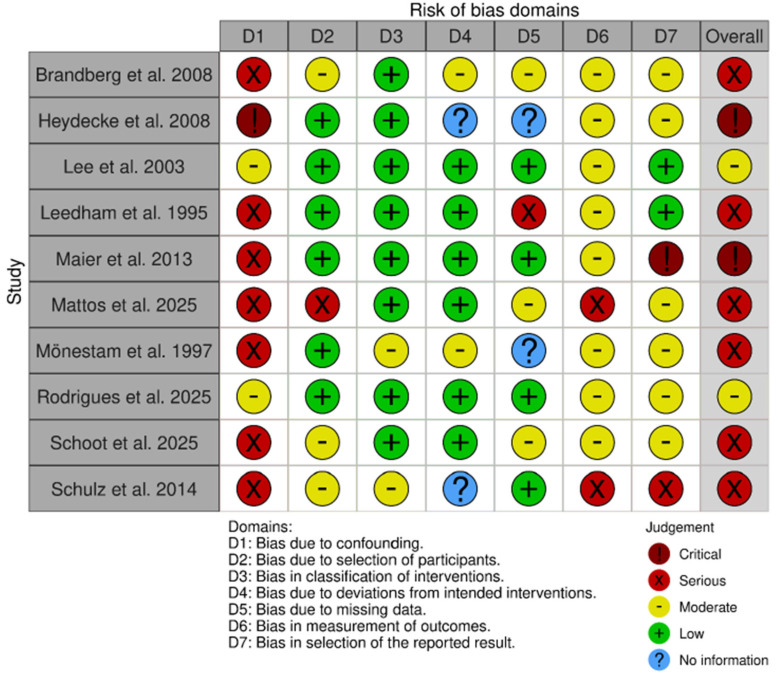
Risk of bias summary. Review authors’ judgements about each risk of bias domain for each included study [24,25,26,27,28,29,30,31,32,33].

ROBINS-I was used to assess the risk of bias in the included cohort studies. Two studies were overall rated as critical [29,30], six as serious [25,27,28,31,33,34], and two as moderate [26,32]. Eight studies [25,27,28,29,30,31,33,34] had high risk of bias, and two had medium risk of bias [26,32] see Figure 2.

## 4. Discussion

The primary outcome correlation between patients’ preoperative expectations and QoL at three and six months postoperatively across various surgical procedures was only reported in two studies [26,29] with divergent results. In general, the findings were heterogeneous and limited by reliance on unvalidated patient expectations tools. Most of the included studies had a high risk of bias, which affects the certainty of the evidence.

### 4.1. Patient Expectations in Nursing Care

Addressing and aligning patients’ preoperative expectations should be an essential part of preparing patients preoperatively in a person-centered way for what to realistically expect from the postoperative course. Expectations aligned prior to surgery may shape patients’ interpretations of symptom relief, functional recovery, and overall quality of life, regardless of objective clinical results [35,36,37]. When expectations are overly optimistic or inadequately addressed, patients may experience dissatisfaction or a reduced perceived benefit, even when surgical outcomes are clinically satisfactory [10]. Conversely, realistic and well-informed expectations may contribute to higher satisfaction and better perceived postoperative quality of life and may reduce regrets related to the surgical procedure [35].

Patient expectations should be revisited in the postoperative period as part of ongoing patient education and again later to prepare the patient for discharge. Actively exploring patients’ perceptions after surgery; for example, by asking “Is this how you expected it to be?” may provide valuable insight into how patients interpret their recovery process and whether their expectations have been met. Such dialogue can help nurses identify recurring mismatches between expectations and outcomes, thereby informing future preoperative patient education and communication strategies.

### 4.2. Healthcare Professionals’ Competencies in Expectation Management

Effective management of PE requires more than the provision of standardized information by healthcare professionals and may benefit from a patient-centered care approach that incorporates soft competencies such as empathetic communication, active listening, shared decision-making, and the ability to elicit and address patients’ concerns, fears, and prior assumptions about surgery and recovery [38]. In particular, the capacity to tailor information to individual patients’ cognitive and emotional needs may help ensure that expectations are both realistic and personally meaningful. Healthcare professionals who are skilled in expectation management are better able to identify discrepancies between patients’ understanding and clinically realistic outcomes, and to adjust communication accordingly. This may contribute to improved psychological preparedness, reduced anxiety, and a more accurate interpretation of postoperative recovery, ultimately influencing both satisfaction and perceived quality of life [39,40]. Conversely, insufficient communication skills or failure to actively engage with patients’ expectations may result in unmet expectations, even in cases of objectively successful surgical outcomes. Developing these competencies should be supported through structured education and training initiatives. This may include formal communication training in undergraduate and postgraduate curricula, simulation-based learning, interdisciplinary workshops, and reflective practice aimed at strengthening clinicians’ ability to manage complex patient interactions [38]. Continuous professional development is particularly important in surgical settings, where time constraints and high clinical workload may otherwise limit in-depth patient communication. From an organizational perspective, expectation management competencies should be recognized as a key component of high-quality surgical care.

### 4.3. Instruments

In this systematic review, the included studies [25,26,27,28,29,30,31,32,33,34] primarily relied on unvalidated instruments to measure preoperative expectations. This could present methodological challenges, as such instruments may lack reliability and validity and may be more susceptible to bias and measurement error, thereby limiting comparability across studies. The use of validated instruments is therefore preferable when assessing patient expectations. One example is the Treatment Expectations Questionnaire (TEX-Q) [41], a validated, generic instrument designed to assess treatment expectations across medical contexts. However, the TEX-Q has not yet been translated and validated in a broad range of languages, which may limit its applicability. Regarding the assessment of postoperative outcomes such as quality of life and patient satisfaction, several of the included studies [25,26,29,30,32,33] used validated instruments, including the SF-36 [25,26], PDQ-39 [29], VAS [30,32], and RTSQ [33], which strengthens the validity of the findings.

This means that results cannot be generalized quantitatively (no precise, robust estimates), though there is some qualitative/general signal that expectations are relevant across diverse non-orthopedic surgeries; however, any generalization should be regarded as hypothesis-generating, requiring confirmation with validated instruments and stronger designs.

### 4.4. Patient Populations

An important limitation regarding generalizability is that more than half of the included patients (605/1013) were transplant recipients. Transplant patients represent a highly specific clinical population characterized by complex preoperative education, intensive follow-up, and distinct postoperative trajectories compared with other surgical groups. Their expectations, informational needs, and determinants of postoperative quality of life may differ substantially from those of patients undergoing other types of surgery. As a result, the overall findings may be partly driven by this subgroup, which limits the extent to which the results can be generalized to broader non-orthopedic surgical populations. Consequently, the findings should be interpreted with caution, and future studies should consider stratified analyses according to surgical subgroups.

### 4.5. Strengths and Limitations

The strength of this review lies in its comprehensive and systematic search across multiple databases. The search strategy, conducted in accordance with the PRISMA guideline [21], ensured a systematic approach to identifying relevant studies, thereby enhancing the transparency and reliability of the review process. However, several limitations should be acknowledged. First, this review was based on only 10 studies [25,26,27,28,29,30,31,32,33,34], which included heterogeneous surgical procedures, study designs, and methods for assessing PE. This clinical and methodological heterogeneity limited comparability across studies and precluded the performance of subgroup analyses or meta-analysis. Notably, most included studies used non-validated or self-constructed instruments to measure expectations, introducing substantial variability in measurement approaches and limiting the reliability, interpretability, and reproducibility of the findings. This lack of validated measures of expectation represents a major methodological limitation. In addition, some studies [25,33,34] did not specifically examine associations between PE and postoperative outcomes, further limiting the ability to draw firm conclusions. Furthermore, no GRADE evaluation was conducted because all included studies were cohort studies and therefore considered to provide low-certainty of evidence. Language restrictions may also have introduced selection bias, as only studies published in English or the Nordic languages were included. In addition, the possibility of publication bias cannot be excluded, as studies reporting non-significant associations may be less likely to be published.

To improve the reliability and comparability of future research, well-designed prospective studies using validated instruments to measure PE, such as the TEX-Q [41], and standardized outcome measures are needed. Unvalidated tools and high risk of bias mean the review’s findings have very limited, low-certainty generalizability. They support only cautious, conceptual generalization—that preoperative expectations seem clinically relevant—while any strong or broadly applicable claims about effect sizes or specific subgroups are not justified and should be framed as provisional and hypothesis-generating rather than definitive.

## 5. Conclusions

This review found limited evidence to support an association between patients’ preoperative expectations and postoperative quality of life. The included studies were heterogeneous in terms of surgical procedures, methodologies, and outcome measures, and most studies used unvalidated instruments to assess PE and were judged to have a high risk of bias. Therefore, the findings should be interpreted with caution.

Although PE may be relevant to perioperative nursing care, the current evidence is insufficient to support definitive clinical recommendations regarding expectation alignment and postoperative outcomes. Nevertheless, facilitating discussions about patient expectations may help support patient communication and shared understanding regarding postoperative recovery, pain management, and rehabilitation goals. Future large-scale trials using validated instruments and standardized outcome measures are needed to better clarify the relationship between preoperative expectations and postoperative quality of life or satisfaction.

## Figures and Tables

**Table 1:** Characteristics of included studies.

Study	Study Design	Number of Participants	Surgical Procedure	Follow-Up Time in Months	Preoperative Expectation	Quality of Life	Satisfaction
Brandberg et al. 2008 [25]	Prospective study	90	Bilateral Prophylactic Mastectomy	6 and 12	Self-constructed, 6-item questionnaire	SF-36	NA
Heydecke et al. 2008 [30]	Cohort	162	Mandibular Implant Overdentures (IOD)	6	Self-constructed, 2-item questionnaire	NA	VAS
Lee et al. 2003 [26]	Prospective cohort	313	Hematopoietic Stem Cell Transplantation	6	Self-constructed, 2-item questionnaire	SF-36	NA
Leedham et al. 1995 [34]	Cohort	31	Heart transplant	3 and 6	Self-constructed, 7-item questionnaire	Self-constructed, 1 item questionnaire	NA
Maier et al. 2013 [29]	Mixed methods	30	Bilateral subthalamic deep brain stimulation	3	Self-constructed qualitative interview	PDQ-39	NA
Mattos et al. 2025 [31]	Mixed methods	52	Sinus surgery	6	Self-constructed questionnaire and interviews covering 11 domains	NA	Self-constructed, 2-item questionnaire
Mönestam et al. 1997 [28]	Prospective cohort	52	Cataract surgery	5 to 6	Self-constructed, 1-item questionnaire	NA	Self-constructed, 1-item questionnaire
Rodrigues et al. 2025 [32]	Observational study	22	Immediate-loaded zygomatic implant	6	Visual Analog Scale, 1–100 mm 5-item questionnaire, self-constructed	NA	Visual Analog Scale, 1–100 mm. Self-constructed, 5-item questionnaire
Schoot et al. 2025 [33]	Prospective cohort	33	Kidney transplant	6	Four items from the SF-12	NA	Renal Treatment Satisfaction Questionnaire
Schulz et al. 2014 [27]	Prospective cohort	228	Kidney transplant	3, 6 and 12	Self-constructed, 3-item questionnaire	Self-constructed, 3-item questionnaire	NA

Note. Overview of included studies, including study design, number of participants, type of surgical procedure, follow-up period, and measurement of preoperative expectations, quality of life (QoL), and patient satisfaction. All expectation measures were self-constructed and non-validated. In Schoot et al. (2025) [33] they rephrased the questions from SF-12; for example, the original question ‘In general, would you say your health is…’ was rephrased as ‘In general, what do you expect your health to be six months after kidney transplantation? The other three questions were according to “limitations in moderate activities”, “limitations in climbing stairs” and “interference with social activities”. QoL = Quality of Life; SF-36 = Short Form Health Survey; SF-12 = The 12-item Short Form Survey; PDQ-39 = Parkinson’s Disease Questionnaire; VAS = Visual Analogue Scale; NA = Not Applicable.

**Table 2:** Overview of results from each included study.

Study	Results
Brandberg et al. 2008 [25]	They found no significant changes in Quality of Life (QoL) across the three assessment points. Additionally, bilateral prophylactic mastectomy showed no negative effects on anxiety, depression, or overall quality of life; in fact, anxiety levels improved. However, there was a negative impact on sexuality and body image [25].
Heydecke et al. 2008 [30]	A significant association was found between preoperative expectations and post-surgery satisfaction for 2-implant-supported mandibular overdentures (IOD) in the Middle-Aged group but not in the Senior group. The authors conclude that patients’ expectations of satisfaction with implant overdentures are very high, but also that this treatment is likely to meet patients’ expectations [30].
Lee et al. 2003 [26]	The study found no significant difference between the optimistic and less optimistic groups and QoL at 6-month follow-up. They found that patients with optimistic expectations about their transplant outcomes had a higher survival rate within the first 60 days compared to those with lower expectations. This association remained significant even after adjusting patients’ physical and mental health status. However, by 6 months, there were no significant differences in survival or QoL between the groups [26].
Leedham et al. 1995 [34]	The findings show that QoL increased immediately after surgery and remained improved at 3- and 6-month follow-up. The authors noted the 6-month follow-up period as a limitation, as a strong relationship between preoperative expectations and later physical health may exist. However, it remains unclear whether expectations would similarly predict outcomes over a longer follow-up period [34].
Maier et al. 2013 [29]	They found no significant difference between groups at baseline for QoL, although the group-negative initially had the poorest QoL. At the 3-month follow-up, QoL scores were significantly higher in the group-negative than in the group-mixed and group-positive. Within-group changes indicated that QoL significantly improved only in the group-mixed and group-positive. Attention! A higher score indicates poorer QoL [29].
Mattos et al. 2025 [31]	According to the mediation analysis conducted they found complete mediation, indicating that clinical outcome no longer affected patient satisfaction with the outcome after controlling for the fulfillment of preoperative expectations. Furthermore, the ratio of indirect and total effect (RIT) was 0.34, suggesting that 34% of the effect of clinical outcome on satisfaction with the outcome could be explained by the fulfillment of preoperative expectations. Furthermore, the *p*-value is less than 0.05 according to the effect of the fulfillment of preoperative expectations on satisfaction with outcome. The authors note that calibrating preoperative expectations appropriately may be the single most important intervention that a sinus surgeon can perform [31].
Mönestam et al. 1997 [28]	They found no association between the level of preoperative expectations and the degree of postoperative satisfaction. However, patient satisfaction increased with better functional outcomes of the operated eye. A statistically significant correlation was found between expectations of improvement and the ‘disability index’, with higher levels of visual impairment associated with higher expectations for surgery. [28].
Rodrigues et al. 2025 [32]	A statistically significant association was found between preoperative expectations and satisfaction with the impact of rehabilitation on speech, aesthetics, chewing, and social life. However, regarding changes in satisfaction over time, only chewing showed a statistically significant association at 6 months post-surgery. The satisfaction levels were not influenced by age and sex as the *p*-value showed no statistical significance. The authors further state that understanding and assessing patient expectations is recommended in clinical encounters, as expectations may influence treatment outcomes [32].
Schoot et al. 2025 [33]	They found that 53% of kidney transplant recipients fully achieved all their personal treatment goals six months after transplantation, while 47% did not. Goals related to improvements in medical, psychological, functional, and social domains were generally fully achieved by approximately 50–60% of patients, partially achieved by 20–35%, and not achieved by 10–25%. Patients frequently overestimated the benefits of transplantation and underestimated the likelihood of complications; for example, 57% did not expect complications, yet only 41% of these patients experienced none. Despite incomplete fulfillment of expectations, overall treatment satisfaction was high, and decision regret was low at six months post-surgery. The authors conclude that these findings may help improve patient education programs and inform shared decision-making processes for patients with kidney failure [33].
Schulz et al. 2014 [27]	Across assessments, significant changes in QoL were noted in all dimensions, for physical, psychological and social QoL. Compared with pre-transplant ratings, QoL increased in all dimensions and improvements were maintained throughout all assessments, except for psychological QoL. The effect sizes were moderate for improvements in physical QoL and small for psychological and social QoL. However, significant differences were also observed between expected and actual QoL ratings for physical, psychological and social QoL. Post-transplant QoL was lower than expected for all dimensions, and this disparity continued throughout all assessments. The magnitude of this effect was moderate for physical QoL and small for psychological QoL and social QoL overestimation. Further research is recommended to investigate the factors contributing to QoL overestimation, including the roles of patient education and individual differences in processing information [27].

Note. This table summarizes key findings from each included study regarding the relationship between patient expectations and postoperative outcomes. A higher QoL score in Maier et al. (2013) [29] indicates poorer quality of life.

**Table 3:** Classification of patients into groups according to expectations.

	Negative/Low	Neutral	Medium	Positive/High
Brandberg et al. 2008 [25]				
Heydecke et al. 2008 [30]				
Lee et al. 2003 [26]	+			+
Leedham et al. 1995 [34]				
Maier et al. 2013 [29]	+	+		+
Mattos et al. 2025 [31]				
Mönestam et al. 1997 [28]				
Rodrigues et al. 2025 [32]	+		+	+
Schoot et al. 2025 [33]				
Schulz et al. 2014 [27]				

Note. A ‘+’ indicates that the study categorized patients into the respective expectation group (negative/low, neutral, medium or positive/high). Most studies did not group patients by expectations. Classification is based on how patients were grouped in each study according to their preoperative expectations.

## Data Availability

The raw data supporting the conclusions of this article will be made available by the authors on request.

## Supplementary Materials

The following supporting information can be downloaded at: https://www.mdpi.com/article/10.3390/ijerph23060804/s1, File S1: Search String; File S2: PRISMA 2020 Checklist. Ref. [21] was cited in the Supplementary Material.
