# Supplementary material for: Preoperative Treatment Expectations and Their Association with Postoperative Quality of Life and Patient Satisfaction in Non-Orthopedic Surgery: A Systematic Review
Source: Int J Environ Res Public Health. 2026 Jun 16;23(6):804. doi: 10.3390/ijerph23060804 (PMC13299401; doi:10.3390/ijerph23060804)
Supplement: Supplementary file 1 [file ijerph-23-00804-s001.zip › Suppl 1.pdf]

File S1

Database	Date last searched	Search terms
Embase	05-01-2026	1 exp general surgery/<br>2 "expectation*".af.<br>3 exp Surgical Procedures, Operative/<br>4 exp "quality of life"/<br>5 exp satisfaction/<br>6 general surgery.mp.<br>7 Surgical Procedures.mp.<br>8 1 or 3 or 6 or 7<br>9 patient expectations.mp.<br>10 2 or 9<br>11 patient satisfaction.mp. or exp patient satisfaction/<br>12 quality of life.mp.<br>13 4 or 5 or 11 or 12<br>14 8 and 10 and 13<br>15 (8 and 10 and 13) not (orthopedic* or orthopaedic* or hip or knee or shoulder or elbow or ankle or spine or spinal or lumbar or cervical or hand or wrist or arthroplasty).mp. [mp=title, abstract, heading word, drug trade name, original title, device manufacturer, drug manufacturer, device trade name, keyword heading word, floating subheading word, candidate term word]
Cochrane Central	05-01-2026	ID Search<br>#1 MeSH descriptor: [Patients] explode all trees<br>#2 patient<br>#3 MeSH descriptor: [General Surgery] explode all trees<br>#4 postoperative*<br>#5 MeSH descriptor: [Hospitalization] explode all trees<br>#6 post surgery<br>#7 MeSH descriptor: [Surgical Procedures, Operative] explode all trees<br>#8 expectation*<br>#9 postoperative<br>#10 #1 or #2<br>#11 orthopedic*<br>#12 orthopaedic*<br>#13 hip<br>#14 knee<br>#15 shoulder<br>#16 elbow<br>#17 ankle<br>#18 spine<br>#19 spinal<br>#20 lumbar

		#21 cervical<br>#22 hand<br>#23 wrist<br>#24 arthroplasty<br>#25 #3 or #4 or #5 or #6 or #7<br>#26 #8 and #10 and #25<br>#27 #11 or #12 or #13 or #14 or #15 or #16 or #17 or #18 or #19 or #20 or #21 or #22 or #23 or #24<br>#28 #26 not #27
CINAHL	05-01-2026	S1 “expectations”<br>S2 “surgery”<br>S3 “surgical procedures”<br>S4 “operation”<br>S5 (MH “Quality of Life+”) OR “quality of life”<br>S6 “satisfaction” OR (MH “Patient Satisfaction+”)<br>S7 “patient expectations”<br>S8 “S1 OR S7”<br>S9 “S2 OR S3 OR S4”<br>S10 “S5 OR S6”<br>S11 “S8 AND S9 AND S10”<br>S12 “(S8 AND S9 AND S10) NOT (orthopedic* or orthopaedic* or hip or knee or shoulder or elbow or ankle or spine or spinal or lumbar or cervical or hand or wrist or arthroplasty)”
PsycINFO	05-01-2026	1 exp "quality of life"/<br>2 exp satisfaction/<br>3 general surgery.mp.<br>4 Surgical Procedures.mp.<br>5 patient expectations.mp.<br>6 quality of life.mp.<br>7 exp Surgical Patients/ or exp Surgery/<br>8 exp Expectations/<br>9 expectations.mp.<br>10 satisfaction.mp.<br>11 exp Surgery/<br>12 surgery.mp.<br>13 3 or 4 or 7 or 11 or 12<br>14 5 or 8 or 9<br>15 1 or 2 or 6 or 10<br>16 13 and 14 and 15<br>17 16 not (orthopedic* or orthopaedic* or hip or knee or shoulder or elbow or ankle or spine or spinal or lumbar or cervical or hand or wrist or arthroplasty).mp. [mp=title, abstract, heading word, table of contents, key concepts, original title, tests & measures, mesh word]
MEDLINE	05-01-2026	1 exp general surgery/<br>2 "expectation*".af.<br>3 exp Surgical Procedures, Operative/<br>4 exp "quality of life"/

		5 exp satisfaction/<br>6 general surgery.mp.<br>7 Surgical Procedures.mp.<br>8 1 or 3 or 6 or 7<br>9 patient expectations.mp.<br>10 2 or 9<br>11 patient satisfaction.mp. or exp patient satisfaction/<br>12 quality of life.mp.<br>13 4 or 5 or 11 or 12<br>14 8 and 10 and 13<br>15 14 not (orthopedic* or orthopaedic* or hip or knee or shoulder or elbow or ankle or spine or spinal or lumbar or cervical or hand or wrist or arthroplasty).mp. [mp=title, book title, abstract, original title, name of substance word, subject heading word, floating sub-heading word, keyword heading word, organism supplementary concept word, protocol supplementary concept word, rare disease supplementary concept word, unique identifier, synonyms, population supplementary concept word, anatomy supplementary concept word]
Google Scholar	05-01-2026	(expectations or patient expectations) and (surgery or general surgery or surgical procedures) and (quality of life or satisfaction)
